# Predictors and prescribing patterns of definite anticholinergic medication use among community-dwelling older adults in Saudi Arabia: a retrospective multicenter cross-sectional study

**DOI:** 10.3389/fmed.2026.1974639

**Published:** 2026-10-13

**Authors:** Khalid A. Alamer, Mohamed A. Albekery, Noha Keshk, Fawaz M. Alotaibi, Ibrahim S. Alhomoud, Ali H. Alwadai, Mansour M. Alotaibi, Ahmad M. Almuslim, Ali H. Nasfan, Munthir Abdulmajeed Aldukhi, Turki Aldossary, Nemer AlMosyab, Khaled S. Alnaim, Wassan A. Alnaim, Yasser A. Alharbi, Mohammed Y. Shabib, Rawan M. Alhulibi, Norah A. Alayesh

**Affiliations:** 1 Department of Pharmacy Practice, College of Pharmacy, Imam Abdulrahman Bin Faisal University, Dammam, Saudi Arabia; 2 Department of Pharmacy Practice, College of Clinical Pharmacy, King Faisal University, Al-Ahsa, Saudi Arabia; 3 Department of Pharmacy Practice, College of Pharmacy, Purdue University, West Lafayette, IN, United States; 4 Department of Pharmacy Practice, Faculty of Pharmacy, Mansoura University, Mansoura, Egypt; 5 Department of Pharmacy Practice, College of Pharmacy, Qassim University, Qassim, Saudi Arabia; 6 Department of Pharmacy Practice, College of Pharmacy, Najran University, Najran, Saudi Arabia; 7 Clinical Trial Unit, Translational Medical Research Center, Imam Abdulrahman Bin Faisal University, Dammam, Saudi Arabia; 8 Department of Psychiatry, Imam Abdulrahman Bin Faisal Hospital, Ministry of National Guard Health Affairs, Dammam, Saudi Arabia

**Keywords:** anticholinergic burden, anticholinergic cognitive burden scale, inappropriate prescribing, medication safety, older adults, polypharmacy, Saudi Arabia

## Abstract

**Background/objectives:**

Anticholinergic medications are associated with cognitive impairment and falls in older adults, yet multicenter evidence from Saudi Arabia remains limited. This study characterized prescribing patterns and factors associated with definite anticholinergic burden among older adults receiving outpatient care.

**Methods:**

This retrospective multicenter cross-sectional study used outpatient records and dispensing data from two tertiary hospitals located in Al Khobar and Al-Ahsa, Saudi Arabia, between 2019 and 2024. Adults aged 60 years or older receiving at least one medication scoring 2 or 3 on the Anticholinergic Cognitive Burden (ACB) scale were included. Robust linear, negative binomial, and logistic regression models examined definite ACB score, additional qualifying medications, and multiple medication use.

**Results:**

The study included 1,664 older adults with 2016 patient-medication instances. Polypharmacy was present in 84.3%, and 17.5% received multiple qualifying medications. Bladder antimuscarinics, antipsychotics, and tricyclic antidepressants accounted for 78.1% of medication instances, while urology, psychiatry, and neurology accounted for 72.0%. A corresponding coded diagnosis was identified for 20.0% of evaluable instances. Polypharmacy, hypertension, depression, Parkinson's disease, schizophrenia, and bipolar disorder were independently associated with all three outcomes. Schizophrenia showed the strongest associations with definite ACB score (*B* = 2.206; 95% CI, 0.429–3.982), additional medications (incidence rate ratio = 4.395; 95% CI, 2.499–7.732), and multiple medication use (adjusted odds ratio = 8.303; 95% CI, 3.275–21.055).

**Conclusions:**

Definite anticholinergic use was concentrated within a limited number of medication classes and prescribing specialties. Psychiatric disorders and polypharmacy were consistently associated with higher burden, supporting targeted medication review in these patients and specialties.

## Introduction

Medications with anticholinergic properties are frequently prescribed among older adults for several medical conditions, including urinary dysfunction, gastrointestinal disorders, and Parkinson's disease (1). However, despite these benefits, medications with strong anticholinergic effects are considered high-risk medications in older adults according to the 2023 American Geriatrics Society Beers Criteria (2) and the Screening Tool of Older Persons' Prescriptions/Screening Tool to Alert to Right Treatment (STOPP/START) version 3 (3) because of their associated adverse effects. The cumulative effect of these medications can be estimated using the Anticholinergic Cognitive Burden (ACB) scale, which ranks medications according to their anticholinergic activity and potential cognitive effects (4).

Higher anticholinergic burden has been associated with reduced physical and cognitive function (5, 6), impaired ability to perform activities of daily living (7), falls (8, 9), dementia (10), and mortality (9). The association with dementia has been observed with exposure occurring years before diagnosis, suggesting potential long-term cognitive effects (10). With the number of people living with dementia projected to increase substantially worldwide, reducing avoidable anticholinergic exposure remains an important medication safety priority (11, 12). Approximately 1.7 million individuals in Saudi Arabia were aged ≥60 years in 2025, equivalent to 4.8% of the total population (13).

Despite the development of several tools to identify potentially inappropriate prescribing (14), recent evidence from the United States indicates that anticholinergic burden remains substantial among older adults, although overall exposure has declined over time (15, 16). A different trend was reported in the United Kingdom, where anticholinergic burden increased up to ninefold over 25 years (17). In Saudi Arabia, data on the prevalence and prescribing patterns of anticholinergic use remain limited and have largely been reported within broader evaluations of potentially inappropriate medications in general older populations or selected clinical groups (18–21). Polypharmacy is also prevalent among older adults in Saudi Arabia (22) and has been consistently associated with greater anticholinergic burden (17, 23, 24).

Understanding the factors associated with a higher burden of definite anticholinergic medication use among older adults in Saudi Arabia is important for identifying patients who may benefit from targeted medication review. Therefore, this multicenter study aimed to characterize prescribing patterns and identify factors associated with definite anticholinergic medication burden among older adults receiving outpatient care.

## Materials and methods

### Study design and setting

This retrospective multicenter cross-sectional study included adults aged 60 years or older with at least one outpatient dispensing record for a medication with an ACB score of 2 or 3. The cutoff of 60 years was selected in line with the age threshold used in Saudi national statistics for older adults (13). The study population was described as older adults receiving outpatient care. Data were obtained from two tertiary hospitals located in Al Khobar and Al-Ahsa in the Eastern Province of Saudi Arabia between 2019 and 2024. The two hospitals represent separate tertiary health systems serving different cities within the Eastern Province.

### Data sources and study variables

At the Al Khobar site, demographic characteristics, coded diagnoses, medications, and prescribing departments were extracted from electronic health record and pharmacy systems. At the Al-Ahsa site, trained abstractors collected the corresponding information from outpatient electronic records. Data were harmonized into a patient-level dataset with one record per patient and a medication-level dataset with one record per unique patient-medication combination. Multiple records for the same medication in the same patient were consolidated into a single patient–medication record.

Patient characteristics included age, sex, marital status, study site, and polypharmacy. Marital status was categorized as married or not married. Polypharmacy was defined as five or more medications recorded in the source data. Medication start and stop dates were not consistently available; therefore, this measure does not confirm concurrent use. Indication-related diagnoses and general comorbidities were identified from International Classification of Diseases, 10th Revision (ICD-10) codes recorded during the study period. Indication-related diagnoses were used for descriptive and concordance analyses but were excluded from regression models because of their direct relationship with the medications contributing to the outcomes.

### Anticholinergic medication classification and outcomes

Medications were classified using the 2012 update of the ACB scale (4, 25). The analysis was restricted to scores 2 and 3 to focus on medications with definite anticholinergic activity; medications with a score of 1 indicate possible activity and were excluded. Qualifying medications were grouped into the following classes: bladder antimuscarinics, antipsychotics, tricyclic antidepressants, selective serotonin reuptake inhibitors, first-generation antihistamines, antiepileptics, antiparkinsonian agents, or other/systemic antimuscarinics. Medication start and stop dates were not consistently available; therefore, temporal overlap between qualifying medications could not be confirmed. The number of anticholinergic medications was the number of distinct qualifying medications recorded for each patient at any time during the study period. Multiple anticholinergic medication use was defined as more than one distinct qualifying medication recorded during the study period. Definite ACB score was calculated by assigning 2 points to each score-2 medication and 3 points to each score-3 medication and summing these values across all distinct qualifying medications for each patient. For the count regression analysis, the number of additional anticholinergic medications was calculated as the total number of qualifying anticholinergic medications minus one because all included patients had at least one qualifying medication.

Coded diagnosis concordance was defined as the presence of at least one prespecified coded diagnosis corresponding to the medication class. Bladder antimuscarinics were matched to overactive bladder or urinary incontinence; antipsychotics to schizophrenia or bipolar disorder; tricyclic and selective serotonin reuptake inhibitor antidepressants to depression or anxiety; first-generation antihistamines to insomnia, allergic rhinitis or urticaria, and nausea, vomiting or vertigo; antiepileptics to seizure disorder or epilepsy; and antiparkinsonian medications to Parkinson's disease. Dementia alone was not considered concordant for antipsychotic use. Other/systemic antimuscarinics were excluded because no single corresponding indication was available. Concordance was interpreted as a coded-documentation measure, not as evidence of prescribing appropriateness. Drug-class co-occurrence was examined among patients receiving multiple anticholinergic medications. Off-diagonal cells represented receipt of medications from both classes, whereas diagonal cells represented receipt of at least two distinct medications within the same class.

### Statistical analysis

Continuous variables were summarized using mean and standard deviation or median and interquartile range. Categorical variables were summarized using frequency and percentage. Patient-level site comparisons used Welch's *t* test or the Mann–Whitney *U* test for continuous variables and Pearson's chi-square test or Fisher's exact test for categorical variables. Sex-stratified comparisons used Welch's *t* test for age, Mann–Whitney *U* tests for skewed continuous variables, and Pearson's chi-square test or Fisher's exact test for categorical variables. Medication-class and prescribing-department distributions were presented descriptively because patients could contribute multiple medication records. Age, sex, marital status, polypharmacy, and study site were retained *a priori* in all multivariable models. The 16 general comorbidities were screened against the three burden outcomes, and those associated with at least one outcome at *p* < 0.10 were retained. These were dementia, hypertension, depression, diabetes mellitus, ischemic cortical infarction, Parkinson's disease, schizophrenia, bipolar disorder, dyslipidemia, and history of falls. The *p* < 0.10 threshold was used to reduce the risk of excluding potentially relevant covariates while limiting the number of variables in the models. A sensitivity analysis included all 16 general comorbidities regardless of their bivariate associations.

Definite ACB score was analyzed using a generalized linear model with a normal distribution and identity link. Heteroskedasticity-robust standard errors were used because the outcome was concentrated and right-skewed. A Gamma model with a log link and robust standard errors was used as a sensitivity analysis. Negative binomial regression was used for the number of additional anticholinergic medications, and logistic regression was used for multiple anticholinergic medication use. Results were reported as unstandardized coefficients, incidence rate ratios, or adjusted odds ratios with 95% confidence intervals. The primary multivariable models included 1,533 complete cases; 131 patients were excluded because sex was missing. Sensitivity analyses repeated all three multivariable models in the full cohort of 1,664 patients after removing sex from the covariate set. Multicollinearity and model fit were assessed using standard diagnostic measures. Site interactions with age, polypharmacy, and depression were examined in the logistic model. Tests were two-sided, with *p* < 0.05 indicating statistical significance. Analyses were conducted using IBM SPSS Statistics for Windows, version 27.0 (IBM Corp., Armonk, NY, USA).

### Ethical approval

The study was approved by the Institutional Review Board of Imam Abdulrahman Bin Faisal University (IRB-2026-05-0472; 22 June 2026). Data collection at the Al-Ahsa site was conducted under approval 0000098725 from King Abdullah International Medical Research Center, registered with the National Committee of Bioethics (H-01-R-005; 2 January 2025).

## Results

The study included 1,664 older adults with at least one qualifying anticholinergic medication, including 1,318 patients from the Al Khobar site and 346 from the Al-Ahsa site. The mean age was 73.28 years (standard deviation, 7.82), and the prevalence of polypharmacy was 84.3%. Patients at the Al-Ahsa site were younger and had a higher prevalence of polypharmacy, hypertension, diabetes mellitus, stroke, and dyslipidemia. Several indication-related diagnoses were more frequently documented at the Al Khobar site (Table 1). Sex-stratified comparisons are presented in Supplementary Table S3. Women were more often not married and had dementia or depression, whereas men more often had stroke or vasculopathy. None of the three anticholinergic burden outcomes differed by sex.

**Table 1:** Demographic and clinical characteristics of older adults prescribed anticholinergic medications, by study site.

Characteristic	Al Khobar (*n* = 1,318)	Al-Ahsa (*n* = 346)	Total (*N* = 1,664)	*p*
Age, years, mean (SD)	73.76 (7.46)	71.47 (8.84)	73.28 (7.82)	<0.001
Sex, *n* (%)				**0.914**
Female	594 (50.0)	172 (49.7)	766 (50.0)	
Male	593 (50.0)	174 (50.3)	767 (50.0)	
Marital status, *n* (%)				**0.315**
Married	1,090 (82.7)	294 (85.0)	1,384 (83.2)	
Not married	228 (17.3)	52 (15.0)	280 (16.8)	
Polypharmacy (≥5 medications), *n* (%)	1,088 (82.5)	315 (91.0)	1,403 (84.3)	<0.001
Indication-related diagnoses, *n* (%)				
Overactive bladder/incontinence	221 (16.8)	11 (3.2)	232 (13.9)	<0.001
Insomnia	48 (3.6)	1 (0.3)	49 (2.9)	0.001
Allergic rhinitis/urticaria	40 (3.0)	2 (0.6)	42 (2.5)	0.010
Nausea, vomiting, or vertigo	210 (15.9)	0 (0.0)	210 (12.6)	<0.001
Seizure disorder/epilepsy	35 (2.7)	3 (0.9)	38 (2.3)	0.047
Gastrointestinal spasm/irritable bowel syndrome	305 (23.1)	10 (2.9)	315 (18.9)	<0.001
Muscle spasm/musculoskeletal pain	327 (24.8)	5 (1.4)	332 (20.0)	<0.001
Chronic pain	60 (4.6)	1 (0.3)	61 (3.7)	<0.001
Anxiety disorder	61 (4.6)	14 (4.0)	75 (4.5)	0.642
General comorbidities, *n* (%)				
Dementia	89 (6.8)	20 (5.8)	109 (6.6)	0.515
Hypertension	176 (13.4)	235 (67.9)	411 (24.7)	<0.001
Depression	83 (6.3)	6 (1.7)	89 (5.3)	<0.001
Diabetes mellitus	261 (19.8)	155 (44.8)	416 (25.0)	<0.001
Atrial fibrillation	41 (3.1)	9 (2.6)	50 (3.0)	0.621
Atherosclerosis	1 (0.1)	1 (0.3)	2 (0.1)	0.373
Stroke	43 (3.3)	28 (8.1)	71 (4.3)	<0.001
Hemorrhagic infarction	4 (0.3)	0 (0.0)	4 (0.2)	0.586
Ischemic cortical infarction	0 (0.0)	23 (6.6)	23 (1.4)	<0.001
Vasculopathy	12 (0.9)	0 (0.0)	12 (0.7)	0.143
Parkinson's disease	49 (3.7)	7 (2.0)	56 (3.4)	0.120
Schizophrenia	21 (1.6)	1 (0.3)	22 (1.3)	0.064
Bipolar disorder	37 (2.8)	10 (2.9)	47 (2.8)	0.934
Dyslipidemia	73 (5.5)	176 (50.9)	249 (15.0)	<0.001
Traumatic brain injury	26 (2.0)	0 (0.0)	26 (1.6)	0.008
History of falls	24 (1.8)	0 (0.0)	24 (1.4)	0.005

Percentages use the site or full-cohort denominator, except sex, which uses available data (*n* = 1,533; 131 values were missing at the Al Khobar site). Continuous variables were compared using Welch's *t* test; categorical variables used Pearson's chi-square test or Fisher's exact test when expected cell counts were <5. SD, standard deviation.

Bold values represent the overall *p*-values for categorical variables with multiple levels.

Most patients received only medications with an ACB score of 3 (91.7%). Overall, 1,373 patients (82.5%) had one qualifying medication, whereas 291 (17.5%) used multiple anticholinergic medications. The median definite ACB score was 3 (interquartile range, 3–3). Multiple medication use and definite ACB score did not differ significantly between sites, although the distribution of ACB severity patterns differed modestly (*p* = .017) (Table 2).

**Table 2:** Anticholinergic medication count, severity pattern, and definite ACB score by study site.

Measure	Al Khobar (*n* = 1,318)	Al-Ahsa (*n* = 346)	Total (*N* = 1,664)	*p*
ACB severity pattern, *n* (%)				**0.017**
Score 3 only	1,196 (90.7)	330 (95.4)	1,526 (91.7)	
Score 2 only	78 (5.9)	12 (3.5)	90 (5.4)	
Both score 2 and score 3	44 (3.3)	4 (1.2)	48 (2.9)	
Number of qualifying anticholinergic medications, *n* (%)				
1	1,095 (83.1)	278 (80.3)	1,373 (82.5)	
2	184 (14.0)	61 (17.6)	245 (14.7)	
3	30 (2.3)	7 (2.0)	37 (2.2)	
4	7 (0.5)	0 (0.0)	7 (0.4)	
≥5	2 (0.2)	0 (0.0)	2 (0.1)	
Multiple anticholinergic medication use (>1), *n* (%)	223 (16.9)	68 (19.7)	291 (17.5)	0.234
Definite ACB score, median (IQR)	3 (3–3)	3 (3–3)	3 (3–3)	0.069

Multiple use was defined as >1 distinct qualifying medication recorded at any time during the study period. ACB severity pattern and multiple use were compared using Pearson's chi-square test; definite ACB score was compared using the Mann–Whitney *U* test. ACB, anticholinergic cognitive burden; IQR, interquartile range.

Bold values represent the overall *p*-values for categorical variables with multiple levels.

The cohort contributed 2,016 distinct patient-medication instances. Bladder antimuscarinics were the most frequently prescribed class (41.1%), followed by antipsychotics (21.9%) and tricyclic antidepressants (15.1%). Bladder antimuscarinics accounted for 60.8% of medication instances at the Al-Ahsa site compared with 35.9% at the Al Khobar site, while antipsychotics and tricyclic antidepressants were more prominent at the Al Khobar site (Table 3). Solifenacin was the most frequently prescribed medication (34.3%), followed by quetiapine (15.0%) and amitriptyline (14.6%) (Supplementary Table S1). Urology accounted for the largest proportion of medication instances (32.6%), followed by psychiatry (26.0%) and neurology (13.4%) (Table 4). Prescribing patterns were closely aligned with department specialty: bladder antimuscarinics predominated in urology, antipsychotics in psychiatry, and tricyclic antidepressants in neurology (Supplementary Table S2).

**Table 3:** Distribution of anticholinergic medication classes overall and by study site.

Medication class	All instances, *n* (%)	ACB score(s)	Al Khobar, *n* (%)	Al-Ahsa, *n* (%)
Bladder antimuscarinic	829 (41.1)	3	573 (35.9)	256 (60.8)
Antipsychotic	441 (21.9)	2, 3	379 (23.8)	62 (14.7)
Tricyclic antidepressant	304 (15.1)	3	300 (18.8)	4 (1.0)
First-generation antihistamine	128 (6.3)	2, 3	75 (4.7)	53 (12.6)
Antiepileptic	119 (5.9)	2	109 (6.8)	10 (2.4)
Antiparkinsonian agent	85 (4.2)	2, 3	78 (4.9)	7 (1.7)
SSRI antidepressant	81 (4.0)	3	52 (3.3)	29 (6.9)
Other/systemic antimuscarinic	29 (1.4)	3	29 (1.8)	0 (0.0)
Total	2,016 (100.0)		1,595 (100.0)	421 (100.0)

Percentages are based on medication instances; one patient could contribute more than one distinct medication. Site distributions are descriptive and were not compared using inferential tests. ACB, anticholinergic cognitive burden; SSRI, selective serotonin reuptake inhibitor.

**Table 4:** Prescribing departments for qualifying anticholinergic medications overall and by study site.

Prescribing department	All instances, *n* (%)	Al Khobar, *n*	Al-Ahsa, *n*
Urology	657 (32.6)	470	187
Psychiatry	523 (26.0)	473	50
Neurology	270 (13.4)	256	14
Internal Medicine	160 (7.9)	142	18
Family Medicine	61 (3.0)	0	61
Neurosurgery	48 (2.4)	47	1
Emergency Department	45 (2.2)	44	1
Obstetrics & Gynecology	40 (2.0)	25	15
Dermatology	36 (1.8)	5	31
Primary Care	34 (1.7)	34	0
Ophthalmology	30 (1.5)	30	0
Ear Nose and Throat	21 (1.0)	21	0
Surgery	16 (0.8)	12	4
Orthopedic Surgery	13 (0.6)	8	5
Anesthesia	11 (0.5)	10	1
Critical Care Unit	11 (0.5)	11	0
Oncology & Hematology	11 (0.5)	0	11
Nephrology	10 (0.5)	0	10
Cardiology	9 (0.4)	6	3
Other	9 (0.4)	0	9
Total evaluable	2,015 (100.0)	1,594	421

One medication instance had a missing prescribing department. Site distributions are descriptive and were not compared using inferential tests because patients could contribute multiple medication record.

A corresponding coded diagnosis was identified for 398 of 1,987 evaluable medication instances (20.0%). Concordance was highest for SSRI antidepressants (42.0%) and antiparkinsonian medications (40.0%). It was lowest for tricyclic antidepressants (10.9%) and antipsychotics (13.8%) (Table 5). Among patients with multiple anticholinergic medication use, same-class combinations were most frequent for bladder antimuscarinics (*n* = 83) and antipsychotics (*n* = 47). The most common cross-class combinations were antipsychotics with antiparkinsonian medications (*n* = 28), bladder antimuscarinics (*n* = 27), or SSRI antidepressants (*n* = 24) (Table 6).

**Table 5:** Concordance between anticholinergic medication classes and corresponding coded diagnoses.

Medication class	Expected coded diagnosis	*n*	Concordant, *n* (%)	Not concordant, *n* (%)
Bladder antimuscarinic	Overactive bladder or urinary incontinence	829	186 (22.4)	643 (77.6)
Antipsychotic	Schizophrenia or bipolar disorder	441	61 (13.8)	380 (86.2)
Tricyclic antidepressant	Depression or anxiety disorder	304	33 (10.9)	271 (89.1)
First-generation antihistamine	Insomnia; allergic rhinitis/urticaria; nausea, vomiting, or vertigo	128	31 (24.2)	97 (75.8)
Antiepileptic	Seizure disorder or epilepsy	119	19 (16.0)	100 (84.0)
Antiparkinsonian agent	Parkinson's disease	85	34 (40.0)	51 (60.0)
SSRI antidepressant	Depression or anxiety disorder	81	34 (42.0)	47 (58.0)
Other/systemic antimuscarinic	Not applicable	29	–	–
Overall, excluding not applicable		1,987	398 (20.0)	1,589 (80.0)

Concordance indicates whether the electronic record contained at least one prespecified coded diagnosis corresponding to the medication class. Dementia alone was not considered concordant for antipsychotic use. This is a documentation measure and should not be interpreted as prescribing appropriateness. SSRI, selective serotonin reuptake inhibitor.

**Table 6:** Co-occurrence of anticholinergic medication classes among patients prescribed more than one anticholinergic medication.

Class	SSRI-AD	TCA-AD	AED	AH	AM-O	APark	APsy	BladAM
SSRI-AD	0	6	3	9	0	1	24	10
TCA-AD	6	2	17	13	0	9	22	19
AED	3	17	0	2	1	3	12	11
AH	9	13	2	2	1	3	18	8
AM-O	0	0	1	1	0	0	0	0
APark	1	9	3	3	0	1	28	2
APsy	24	22	12	18	0	28	47	27
BladAM	10	19	11	8	0	2	27	83

Off-diagonal cells show patients with at least one medication from both classes. Diagonal cells show patients with at least two distinct medications within the same class. AED, antiepileptic; AH, first-generation antihistamine; AM-O, other/systemic antimuscarinic; APark, antiparkinsonian; APsy, antipsychotic; BladAM, bladder antimuscarinic; SSRI-AD, selective serotonin reuptake inhibitor antidepressant; TCA-AD, tricyclic antidepressant.

In bivariate analyses, dementia, hypertension, depression, diabetes mellitus, ischemic cortical infarction, Parkinson's disease, schizophrenia, bipolar disorder, dyslipidemia, and history of falls were associated with at least one anticholinergic burden outcome. Age was not associated with definite ACB score, the number of additional anticholinergic medications, or multiple medication use (Table 7).

**Table 7:** Bivariate associations of age and comorbidities with anticholinergic burden outcomes.

Characteristic	definite ACB score, *p*	Number of additional anticholinergic medications, *p*	Multiple anticholinergic use, *p*
Age	0.195	0.423	0.426
Dementia	0.002	0.005	0.004
Hypertension	<0.001	<0.001	<0.001
Depression	<0.001	<0.001	<0.001
Diabetes mellitus	0.071	0.050	0.048
Atrial fibrillation	0.595	0.418	0.394
Atherosclerosis	0.688	0.517	1.000
Stroke	0.299	0.242	0.253
Hemorrhagic infarction	0.589	0.721	0.537
Ischemic cortical infarction	0.019	0.038	0.046
Vasculopathy	0.729	0.392	0.704
Parkinson’s disease	0.032	0.001	<0.001
Schizophrenia	<0.001	<0.001	<0.001
Bipolar disorder	<0.001	<0.001	<0.001
Dyslipidemia	<0.001	<0.001	<0.001
Traumatic brain injury	0.361	0.399	0.436
History of falls	0.002	0.002	0.004

Age was assessed using Spearman's rank correlation for definite ACB score and the number of additional anticholinergic medications and the Mann–Whitney *U* test for multiple use. Binary comorbidities were assessed using the Mann–Whitney *U* test for the two numeric outcomes and Pearson's chi-square test or Fisher's exact test for multiple use. General comorbidities associated with at least one outcome at *p* < 0.10 were retained for multivariable modeling. ACB, anticholinergic cognitive burden.

In the adjusted robust linear model, higher definite ACB score was associated with polypharmacy (*B* = 0.432; *p* < 0.001), hypertension (*B* = 0.255; *p* = 0.017), depression (*B* = 0.872; *p* = 0.001), Parkinson's disease (*B* = 0.454; *p* = 0.040), schizophrenia (*B* = 2.206; *p* = 0.015), and bipolar disorder (*B* = 0.918; *p* = 0.007). Study site was not independently associated with definite ACB score (Table 8).

**Table 8:** Factors associated with definite anticholinergic cognitive burden score: multivariable robust linear regression.

Variable	*B*	Robust SE	95% CI	*p*
Age, years	0.001	0.0048	−0.008 to 0.011	0.793
Male sex	−0.036	0.0788	−0.190 to 0.119	0.649
Not married	−0.145	0.0944	−0.330 to 0.040	0.125
Polypharmacy	0.432	0.0708	0.294 to 0.571	<0.001
Al-Ahsa site	−0.112	0.1036	−0.315 to 0.091	0.281
Dementia	0.323	0.1682	−0.006 to 0.653	0.055
Hypertension	0.255	0.1070	0.046 to 0.465	0.017
Depression	0.872	0.2519	0.378 to 1.365	0.001
Diabetes mellitus	0.049	0.1061	−0.159 to 0.257	0.642
Ischemic cortical infarction	0.299	0.2964	−0.282 to 0.880	0.313
Parkinson’s disease	0.454	0.2207	0.021 to 0.886	0.040
Schizophrenia	2.206	0.9064	0.429 to 3.982	0.015
Bipolar disorder	0.918	0.3376	0.257 to 1.580	0.007
Dyslipidemia	0.186	0.1328	−0.074 to 0.446	0.161
History of falls	1.213	0.8181	−0.391 to 2.816	0.138
Constant	2.887	0.3557	2.190 to 3.584	<0.001

The complete-case model included 1,533 patients. The model used a normal distribution with identity link and heteroskedasticity-robust sandwich standard errors. Reference categories were female, married, no polypharmacy, and the Al Khobar site. Likelihood-ratio chi-square = 159.303, df = 15, *p* < 0.001. Conventional *R*^2^ = 0.099 and adjusted *R*^2^ = 0.090 are provided as descriptive fit indices. Maximum variance inflation facto*r* = 1.612. CI, confidence interval; SE, standard error.

In the negative binomial model, schizophrenia showed the strongest association with the number of additional qualifying anticholinergic medications (incidence rate ratio [IRR] = 4.395; 95% confidence interval [CI], 2.499–7.732). Higher medication counts were also associated with polypharmacy, hypertension, depression, Parkinson's disease, bipolar disorder, and history of falls. No independent association was observed for age, sex, or study site (Table 9).

**Table 9:** Factors associated with the number of additional anticholinergic medications: multivariable negative binomial regression.

Variable	*B*	IRR	95% CI for IRR	*p*
Age, years	−0.002	0.998	0.982–1.015	0.844
Male sex	−0.104	0.901	0.712–1.142	0.389
Not married	−0.323	0.724	0.506–1.036	0.077
Polypharmacy	1.166	3.210	1.842–5.594	<0.001
Al-Ahsa site	−0.236	0.790	0.551–1.132	0.198
Dementia	0.378	1.459	0.970–2.195	0.070
Hypertension	0.408	1.503	1.108–2.040	0.009
Depression	0.910	2.484	1.722–3.582	<0.001
Diabetes mellitus	0.062	1.064	0.813–1.392	0.653
Ischemic cortical infarction	0.296	1.344	0.604–2.992	0.469
Parkinson’s disease	0.656	1.927	1.185–3.136	0.008
Schizophrenia	1.481	4.395	2.499–7.732	<0.001
Bipolar disorder	0.893	2.443	1.532–3.897	<0.001
Dyslipidemia	0.228	1.256	0.880–1.793	0.209
History of falls	0.829	2.292	1.256–4.181	0.007
Intercept	−2.793	0.061	0.016–0.236	<0.001

The complete-case model included 1,533 patients. The outcome was the number of distinct qualifying medications minus one. Reference categories were female, married, no polypharmacy, and the Al Khobar site. Likelihood-ratio chi-square = 125.717, df = 15, *p* < 0.001; Pearson chi-square/df = 1.011; Akaike information criterion = 1,651.833. The estimated dispersion parameter was 0.429 (95% CI, 0.193–0.953). CI, confidence interval; IRR, incidence rate ratio.

Similarly, schizophrenia showed the strongest association with multiple anticholinergic medication use (adjusted odds ratio [AOR] = 8.303; 95% CI, 3.275–21.055). Polypharmacy (AOR = 3.158), depression (AOR = 3.030), bipolar disorder (AOR = 2.837), Parkinson's disease (AOR = 2.781), history of falls (AOR = 2.640), dementia (AOR = 1.846), and hypertension (AOR = 1.772) were also independently associated with multiple use. Study site was not associated with this outcome. The logistic model showed acceptable discrimination (area under the receiver operating characteristic curve = 0.720) (Table 10). Full-cohort sensitivity analyses excluding sex produced materially similar findings across all three multivariable models. The Gamma model produced similar results. Polypharmacy, hypertension, depression, Parkinson's disease, schizophrenia, and bipolar disorder remained associated with higher definite ACB score; dementia was also associated with this outcome. Schizophrenia showed the strongest association (adjusted mean ratio = 1.593; 95% CI, 1.214–2.091). Models including all 16 comorbidities also produced materially similar findings. Estimates for rare conditions were unstable and were not interpreted.

**Table 10:** Factors associated with multiple anticholinergic medication use: multivariable logistic regression.

Variable	*B*	AOR	95% CI for AOR	*p*
Age, years	−0.001	0.999	0.979–1.019	0.914
Male sex	−0.144	0.866	0.653–1.146	0.314
Not married	−0.416	0.660	0.435–1.002	0.051
Polypharmacy	1.150	3.158	1.765–5.652	<0.001
Al-Ahsa site	−0.217	0.805	0.532–1.219	0.306
Dementia	0.613	1.846	1.123–3.034	0.016
Hypertension	0.572	1.772	1.233–2.548	0.002
Depression	1.108	3.030	1.863–4.927	<0.001
Diabetes mellitus	−0.022	0.978	0.708–1.351	0.892
Ischemic cortical infarction	0.586	1.796	0.708–4.560	0.218
Parkinson’s disease	1.023	2.781	1.514–5.107	0.001
Schizophrenia	2.117	8.303	3.275–21.055	<0.001
Bipolar disorder	1.043	2.837	1.476–5.452	0.002
Dyslipidemia	0.309	1.362	0.893–2.077	0.152
History of falls	0.971	2.640	1.088–6.409	0.032
Constant	−2.834	0.059		<0.001

Multiple use was defined as >1 distinct qualifying medication recorded at any time during the study period. The complete-case model included 1,533 patients, including 274 events. Reference categories were female, married, no polypharmacy, and the Al Khobar site. Likelihood-ratio chi-square = 124.586, df = 15, *p* < 0.001; Hosmer–Lemeshow *p* = 0.078; Nagelkerke *R*^2^ = 0.128; area under the receiver operating characteristic curve = 0.720 (95% CI, 0.688–0.752). Full-cohort sensitivity analyses excluding sex produced materially similar estimates across all three models; site interactions with age, polypharmacy, and depression were not significant. AOR, adjusted odds ratio; CI, confidence interval.

## Discussion

This multicenter study assessed prescribing patterns and factors associated with definite anticholinergic medication burden among older adults receiving outpatient care in Saudi Arabia. Several clinical conditions were associated with higher definite anticholinergic burden, with the strongest associations observed for psychiatric disorders. Depression, schizophrenia, and bipolar disorder were associated with all three burden outcomes. Schizophrenia showed the strongest association with both the number of additional anticholinergic medications and multiple medication use, while polypharmacy, hypertension, and Parkinson's disease were also consistently associated across the three models. Dementia was associated only with multiple medication use, whereas history of falls was associated with the number of additional medications and multiple use. These differences suggest that the associated factors varied across the definite ACB score, number of additional anticholinergic medications, and multiple medication use outcomes. The prominence of psychiatric disorders is clinically plausible because several antidepressants and antipsychotics contribute directly to anticholinergic burden, while insomnia and anxiety are also common in psychiatric populations (1, 26, 27). These findings are consistent with regional studies reporting substantial anticholinergic burden and potentially inappropriate medication use among older adults with psychiatric disorders (19, 28, 29). The association with schizophrenia may reflect antipsychotic use, adjunctive treatment of extrapyramidal symptoms, and psychotropic polypharmacy. However, schizophrenia was documented in only 22 patients, and the estimates had wide confidence intervals. This finding should therefore be interpreted cautiously. Study site was not independently associated with any of the three outcomes after adjustment.

Our study findings also showed that polypharmacy was significantly associated with higher definite anticholinergic burden, a higher number of additional anticholinergic medications, and increased odds of multiple anticholinergic medication use. These associations are consistent with previous studies reporting that patients exposed to a greater number of medications are more likely to have higher anticholinergic burden (17, 23, 24). Given this consistent association, polypharmacy represents a practical marker for identifying older adults who may benefit from targeted medication review. This is especially relevant because polypharmacy is common among older adults in Saudi Arabia (22). Higher anticholinergic burden has been associated with cognitive impairment, delirium, functional decline, and mortality (7, 9, 30, 31). Therefore, comprehensive medication review and deprescribing of potentially inappropriate medications, when clinically appropriate, should be considered important components of care for older adults with polypharmacy (32, 33). Pharmacist-led approaches have reduced anticholinergic exposure in primary care pilot models, and the Reducing the Risk of Dementia through Deprescribing trial is evaluating whether anticholinergic deprescribing improves cognition and safety among community-dwelling older adults (34, 35).

In our cohort, the largest contributors to anticholinergic burden were bladder antimuscarinics, accounting for 41.1% of medication instances, followed by antipsychotics at 21.9% and tricyclic antidepressants at 15.1%. Together, these three classes accounted for 78.1% of all definite anticholinergic medication instances. At the specialty level, urology accounted for 32.6% and psychiatry for 26.0% of medication instances, together representing 58.6%, while neurology contributed a further 13.4%. This concentration within a small number of therapeutic classes and specialties suggests that medication review and prescribing optimization within these areas may be high-yield strategies for reducing avoidable anticholinergic exposure. A similar concentration by medication class and specialty has been reported in the United States, where antidepressants, bladder antimuscarinics, and antihistamines accounted for more than 70% of high-risk anticholinergic prescriptions, with psychiatry and urology among the associated prescribing specialties (36). These findings are also consistent with Saudi studies identifying antipsychotics and antidepressants as prominent potentially inappropriate medications among older psychiatric patients and documenting solifenacin and amitriptyline among anticholinergic medications prescribed to older adults (19, 20, 37). This concentration is clinically relevant because long-term observational evidence has associated dementia risk with exposure to anticholinergic antidepressants, antipsychotics, antiparkinsonian medications, and bladder antimuscarinics (38, 39). Evidence also suggests that neurocognitive effects differ among individual bladder antimuscarinics, with greater concern for oxybutynin and tolterodine and more mixed findings for solifenacin (40, 41).

This study has several limitations. First, medication indications were incompletely documented within the available data. A corresponding coded diagnosis was identified for only 20.0% of evaluable medication instances. However, this measure reflects coded documentation rather than prescribing appropriateness and cannot be used to estimate deprescribing opportunities. More complete documentation and structured medication reconciliation could support future medication review and deprescribing strategies (34). Second, anticholinergic exposure was estimated using the ACB scoring system, which does not account for dose or duration. The available records also did not establish adherence or temporal overlap between medications. Therefore, the definite ACB score represents the sum of scores for distinct qualifying medications recorded at any time during the study period rather than concurrent or cumulative dose-based exposure. Future studies could incorporate measures such as total standardized daily doses to provide a more precise assessment of cumulative exposure (42, 43). Over-the-counter medications with anticholinergic properties were also not captured, which may have resulted in underestimation of overall exposure. This is relevant because direct home medication inventories have identified possession and recent use of over-the-counter anticholinergic medications among community-dwelling older adults (44). Third, diagnoses and medication-diagnosis concordance were based on ICD-10 codes; therefore, misclassification due to incomplete or inaccurate coding cannot be excluded. Data collection also differed by site, with system extraction at Al Khobar and record abstraction at Al-Ahsa. These differences may have influenced the recording of diagnoses and comorbidities despite data harmonization. Both sites were tertiary hospitals in the Eastern Province, which limits generalizability to other regions and care settings. Residential status was not available, so institutional residence could not be excluded. Finally, the cohort included only patients already receiving at least one qualifying anticholinergic medication and cannot estimate prevalence in the broader older population. The cross-sectional design also prevents assessment of temporal relationships. Therefore, the observed associations should be interpreted as correlational rather than causal.

This study has several strengths. Unlike previous Saudi studies that primarily focused on potentially inappropriate medication use, this study characterized therapeutic classes, prescribing specialties, coded medication-diagnosis concordance, and clinical factors associated with three measures of definite anticholinergic burden (18–20). Another key strength is the multicenter design, which included two tertiary hospitals and captured variation in prescribed therapeutic classes and prescribing specialties.

Medication review in older adults with psychiatric disorders should confirm the indication for each psychotropic medication, assess adjunctive anticholinergic therapy and physical comorbidities, and consider gradual deprescribing when appropriate. The available data did not allow assessment of whether physical comorbidities were diagnosed or treated promptly. Recent Saudi evidence also identified the need for clearer deprescribing guidance, integrated workflows, and defined pharmacist roles (45). A Saudi study found that antipsychotic prescribing without a documented qualifying diagnosis was common among older outpatients, further supporting systematic indication documentation and targeted prescribing stewardship (46).

## Conclusion

Psychiatric disorders and polypharmacy were consistently associated with higher definite anticholinergic burden among older adults receiving outpatient care. Anticholinergic prescribing was concentrated within urology, psychiatry, and neurology, with bladder antimuscarinics, antipsychotics, and tricyclic antidepressants accounting for most medication instances. These findings support targeted medication review among older adults with polypharmacy or psychiatric disorders and within the specialties contributing most to anticholinergic exposure. Deprescribing should be individualized and considered when the expected benefits of continued treatment no longer outweigh its potential risks.

## Data Availability

The data analyzed in this study is subject to the following licenses/restrictions: the data are available from the corresponding author upon reasonable request. The data are not publicly available due to patient privacy and institutional restrictions. Requests to access these datasets should be directed to Khalid A. Alamer (kaalamer@iau.edu.sa).
