# Supplementary material for: Predictors and prescribing patterns of definite anticholinergic medication use among community-dwelling older adults in Saudi Arabia: a retrospective multicenter cross-sectional study
Source: Front Med (Lausanne). 2026 Oct 13;13:1974639. doi: 10.3389/fmed.2026.1974639 (PMC13710337; doi:10.3389/fmed.2026.1974639)
Supplement: Supplementary file 1 [file Table1.docx]

**Supplementary Table S1. Anticholinergic medications included in the analysis, with class, score, and frequency**

| **Medication (generic)** | **Medication class** | **ACB score** | **n** | **% of instances** |
| --- | --- | --- | --- | --- |
| Solifenacin | Bladder Antimuscarinic | 3 | 692 | 34.3 |
| Quetiapine | Antipsychotic | 3 | 303 | 15.0 |
| Amitriptyline | Antidepressant (TCA) | 3 | 294 | 14.6 |
| Olanzapine | Antipsychotic | 3 | 131 | 6.5 |
| Trospium | Bladder Antimuscarinic | 3 | 119 | 5.9 |
| Carbamazepine | Antiepileptic | 2 | 119 | 5.9 |
| Paroxetine | Antidepressant (SSRI) | 3 | 81 | 4.0 |
| Promethazine | Antihistamine (1st-gen) | 3 | 67 | 3.3 |
| Benztropine | Antiparkinsonian | 3 | 62 | 3.1 |
| Hydroxyzine | Antihistamine (1st-gen) | 3 | 42 | 2.1 |
| Atropine | Antimuscarinic - Other/Systemic | 3 | 29 | 1.4 |
| Amantadine | Antiparkinsonian | 2 | 12 | 0.6 |
| Oxybutynin | Bladder Antimuscarinic | 3 | 11 | 0.5 |
| Trihexyphenidyl | Antiparkinsonian | 3 | 11 | 0.5 |
| Clomipramine | Antidepressant (TCA) | 3 | 8 | 0.4 |
| Cyproheptadine | Antihistamine (1st-gen) | 2 | 7 | 0.3 |
| Tolterodine | Bladder Antimuscarinic | 3 | 7 | 0.3 |
| Diphenhydramine | Antihistamine (1st-gen) | 3 | 6 | 0.3 |
| Meclizine | Antihistamine (1st-gen) | 3 | 5 | 0.2 |
| Clozapine | Antipsychotic | 3 | 4 | 0.2 |
| Chlorpromazine | Antipsychotic | 3 | 2 | 0.1 |
| Imipramine | Antidepressant (TCA) | 3 | 2 | 0.1 |
| Thioridazine | Antipsychotic | 3 | 1 | 0.0 |
| Dimenhydrinate | Antihistamine (1st-gen) | 3 | 1 | 0.0 |
| **Total** |  |  | **2,016** | **100.0** |

*Note.* Percentages are based on 2,016 distinct patient-medication instances. ACB, Anticholinergic Cognitive Burden.

**Supplementary Table S2. Distribution of anticholinergic medication classes across prescribing departments**

| **Department** | **SSRI-AD** | **TCA-AD** | **AED** | **AH** | **AM-O** | **APark** | **APsy** | **BladAM** | **Total** |
| --- | --- | --- | --- | --- | --- | --- | --- | --- | --- |
| Urology | 5 | 2 | 1 | 0 | 0 | 1 | 0 | 648 | 657 |
| Psychiatry | 11 | 36 | 7 | 24 | 0 | 27 | 416 | 2 | 523 |
| Neurology | 1 | 142 | 66 | 3 | 0 | 50 | 2 | 6 | 270 |
| Internal Medicine | 53 | 47 | 14 | 6 | 0 | 4 | 3 | 33 | 160 |
| Family Medicine | 9 | 0 | 1 | 9 | 0 | 1 | 9 | 32 | 61 |
| Neurosurgery | 0 | 28 | 16 | 0 | 0 | 0 | 0 | 4 | 48 |
| Emergency Department | 0 | 2 | 2 | 34 | 0 | 1 | 1 | 5 | 45 |
| Obstetrics & Gynecology | 1 | 0 | 0 | 0 | 0 | 0 | 0 | 39 | 40 |
| Dermatology | 0 | 1 | 0 | 34 | 0 | 0 | 0 | 1 | 36 |
| Primary Care | 0 | 4 | 3 | 1 | 0 | 0 | 0 | 26 | 34 |
| Ophthalmology | 0 | 1 | 0 | 0 | 29 | 0 | 0 | 0 | 30 |
| Ear Nose and Throat | 0 | 20 | 0 | 0 | 0 | 0 | 0 | 1 | 21 |
| Surgery | 0 | 1 | 5 | 1 | 0 | 0 | 0 | 9 | 16 |
| Orthopedic Surgery | 0 | 3 | 0 | 4 | 0 | 0 | 0 | 6 | 13 |
| Anesthesia | 0 | 9 | 0 | 1 | 0 | 0 | 1 | 0 | 11 |
| Critical Care Unit | 0 | 6 | 3 | 0 | 0 | 1 | 0 | 1 | 11 |
| Oncology & Hematology | 0 | 0 | 0 | 5 | 0 | 0 | 3 | 3 | 11 |
| Nephrology | 1 | 0 | 0 | 2 | 0 | 0 | 3 | 4 | 10 |
| Cardiology | 0 | 1 | 1 | 0 | 0 | 0 | 0 | 7 | 9 |
| Other | 0 | 1 | 0 | 3 | 0 | 0 | 3 | 2 | 9 |
| **Total evaluable** | **81** | **304** | **119** | **127** | **29** | **85** | **441** | **829** | **2015** |

*Note.* One medication instance had a missing prescribing department. AED, antiepileptic; AH, first-generation antihistamine; AM-O, other/systemic antimuscarinic; APark, antiparkinsonian; APsy, antipsychotic; BladAM, bladder antimuscarinic; SSRI-AD, selective serotonin reuptake inhibitor antidepressant; TCA-AD, tricyclic antidepressant.

**Supplementary Table S3. Sex-stratified patient characteristics and definite anticholinergic burden outcomes**

| **Characteristic** | **Female (n = 766)** | **Male (n = 767)** | **p** |
| --- | --- | --- | --- |
| Age, years, mean (SD) | 73.76 (7.65) | 73.41 (7.14) | 0.356 |
| Not married, n (%) | 180 (23.5) | 70 (9.1) | <0.001 |
| Al-Ahsa site, n (%) | 172 (22.5) | 174 (22.7) | 0.914 |
| Polypharmacy, n (%) | 659 (86.0) | 653 (85.1) | 0.618 |
| Total medications, median (IQR) | 14 (8-24) | 14 (7-23) | 0.686 |
| Dementia, n (%) | 71 (9.3) | 38 (5.0) | 0.001 |
| Hypertension, n (%) | 209 (27.3) | 202 (26.3) | 0.675 |
| Depression, n (%) | 55 (7.2) | 34 (4.4) | 0.021 |
| Diabetes mellitus, n (%) | 213 (27.8) | 203 (26.5) | 0.555 |
| Atrial fibrillation, n (%) | 29 (3.8) | 21 (2.7) | 0.248 |
| Atherosclerosis, n (%) | 2 (0.3) | 0 (0.0) | 0.250 |
| Stroke, n (%) | 26 (3.4) | 45 (5.9) | 0.021 |
| Hemorrhagic infarction, n (%) | 3 (0.4) | 1 (0.1) | 0.374 |
| Ischemic cortical infarction, n (%) | 10 (1.3) | 13 (1.7) | 0.531 |
| Vasculopathy, n (%) | 1 (0.1) | 11 (1.4) | 0.004 |
| Parkinson's disease, n (%) | 27 (3.5) | 29 (3.8) | 0.789 |
| Schizophrenia, n (%) | 10 (1.3) | 12 (1.6) | 0.670 |
| Bipolar disorder, n (%) | 29 (3.8) | 18 (2.3) | 0.102 |
| Dyslipidemia, n (%) | 129 (16.8) | 120 (15.6) | 0.526 |
| Traumatic brain injury, n (%) | 17 (2.2) | 9 (1.2) | 0.113 |
| History of falls, n (%) | 13 (1.7) | 11 (1.4) | 0.678 |
| Qualifying anticholinergic medications, median (IQR) | 1 (1-1) | 1 (1-1) | 0.162 |
| Multiple qualifying anticholinergic medication use, n (%) | 147 (19.2) | 127 (16.6) | 0.179 |
| Definite Anticholinergic Cognitive Burden score, median (IQR) | 3 (3-3) | 3 (3-3) | 0.288 |
